# Genetic and antigenic characterization of influenza A(H3N2) in Cameroon during the 2014-2016 influenza seasons

**DOI:** 10.1371/journal.pone.0184411

**Published:** 2017-09-06

**Authors:** Gwladys C. Monamele, Marie-Astrid Vernet, Mohammed R. Njankouo, Kathleen Victoir, Jane Francis Akoachere, Damian Anong, Richard Njouom

**Affiliations:** 1 National Influenza Centre, Centre Pasteur du Cameroun, Yaoundé, Cameroon; 2 Department of Microbiology and Parasitology, University of Buea, Buea, Cameroon; 3 International Director, Institut Pasteur de Paris, Paris, France; The Scripps Research Institute, UNITED STATES

## Abstract

The first outbreak of influenza A(H3N2) occurred in 1968 and caused the third flu pandemic of the 20^th^ century. It has affected multiple countries over time. The best strategy to reduce the burden of influenza is through vaccination whose efficacy varies with respect to the circulating strains. This study was performed to better understand the molecular evolution of influenza A(H3N2) and assess vaccine efficacy in Cameroon. Complete sequences of three gene segments were obtained from 2014 to 2016 influenza seasons in Cameroon. Hemagglutinin (HA), Neuraminidase (NA) and matrix (M) genes of 35 A(H3N2) virus strains were amplified and sequenced. Predicted vaccine efficacy was measured using the P_epitope_ model. Phylogenetic analysis of the HA gene showed that all Cameroonian strains had evolved away from the 3C.1-A/Texas/50/2012-like clade. Globally, 2014 virus strains clustered with the 2015–2016 vaccine strain, 3C.3a-A/Switzerland/9715293/2013, whereas 2015 and 2016 virus strains clustered with the 2016–2017 vaccine strain, 3C.2a-A/HongKong/4801/2014. In order to determine the genotypic drug susceptibility to neuraminidase inhibitors and amantadine, the NA and M2 protein coding sequences were analyzed. There was no strain with characteristic mutation for resistance to neuraminidase inhibitors, per contra; all strains possessed the substitution S31N, peculiar of resistance to adamantanes. There was drift in influenza A(H3N2) dominant epitopes B (2014 and 2015) to epitopes A (2016) with a theoretical efficiency in vaccine ranging from low to moderate. The presence of several antigenic site mutations among H3N2 virus strains between 2014–2016 influenza seasons in Cameroon confirms the progressing evolution of circulating H3N2 strains.

## Introduction

Influenza A virus is a major cause of acute respiratory disease in humans and is responsible for approximately 250,000–500,000 deaths annually worldwide [1]. Pandemic influenza A virus infection resulted in significant morbidity and mortality in 1918 (H1N1), 1957 (H2N2), 1968 (H3N2), and 2009 (H1N1) [2, 3]. The first outbreak of influenza A/H3N2 was reported in 1968 and has since affected multiple countries[2]. In several seasons, A(H3N2) strains are more prevalent than the other co-circulating viral sub-types [4, 5] with high morbidity and mortality rates [6, 7].

Influenza A nomenclature is based on the genotypic properties of two surface glycoproteins, hemagglutinin (HA) and neuraminidase (NA). Currently, 18 HA and 11 NA subtypes have been recognized [8]. The primary target of host neutralizing antibodies is the HA glycoprotein where it inhibits binding to the sialic acid receptors present in the respiratory tract [9]. There are five identified antigenic sites for A(H3N2), namely, A, B, C, D and E [10]. Non-recognition of some strains by neutralizing antibodies present in the host is the consequence of mutations at these sites [11]. Continuous build-up of mutations at these antigenic sites is the basis for the evolutionary dynamics observed in the influenza virus resulting in antigenic drift [12]. About seven A(H3N2) clades (designated clades 1 to 7) and many sub-clades have evolved over the past few years, providing a significant challenge for the annual design of effective influenza vaccines [13]. At present, viruses in clade 3 are the dominant group, which has diversified further into three major subclades designated 3A, 3B, and 3C, with subclade 3C containing several distinguishable genetic groups [13, 14].

The best strategy to reduce the burden of influenza is through vaccination whose efficacy varies with respect to the circulating strains[15]. Although influenza vaccination is very limited in most of Africa, particularly Sub-Saharan Africa, there have been previous reports of introduction of viruses that have undergone drift due to antiviral and vaccine pressure [16, 17] from other regions. There is therefore, a crucial need for monitoring the genetic and antigenic characteristics as well as drug susceptibility of influenza viruses as a component of pandemic preparedness [16]. To better understand the molecular evolution of influenza and assess vaccine efficacy in Cameroon, three gene segments of A(H3N2) from 2014 to 2016 were characterized.

## Materials and methods

### Sample collection and preparation

Respiratory samples were collected between January 2014 and June 2016 from outpatients with clinical evidence of influenza-like illness (ILI), defined as person with sudden onset of fever >38°C and cough or sore throat, at 12 sentinel sites involved in influenza surveillance in Cameroon. Naso-pharyngeal and/or oropharyngeal swabs were collected and put in 2 ml cryovials containing virus transport medium and stored at 4°C before transportation to the Pasteur Centre of Cameroon (PCC) for analysis. All samples were stored anonymously. This study was specifically approved by the Cameroon National Ethics committee (N° 2016/08/798/CE/CNERSH/SP).

Viral RNA was extracted then from 140 μL of clinical samples with Qiagen RNA kit according to the manufacturer’s instructions into a final volume of 60 μL elution buffer. Extracts were analyzed for the presence of influenza by a real-time reverse transcription-polymerase chain reaction (RT-PCR) in an ABI Prism 7300 or 7500 thermocycler (Applied Biosystems, Foster City, California, USA). Positive samples for influenza H3N2 with Ct below 30 after sub-typing were eligible for sequencing. A convenient sample size of 35 was randomly selected from the flu database based on their geographic origin and distribution over time. Samples co-infected with other influenza subtypes were not included.

### Amplification

The first PCR was performed using Superscript III One Step RT-PCR System (Invitrogen, Carlsbad, USA) and a set of primers (S1 Table). The following procedure was used for amplification of 1192 bp of HA gene, 1459bp of NA gene and 1027 bp of M gene of influenza A(H3N2). Overall a total reaction volume of 25 μL contained the following reagents: 12.5 μL of 2x PCR buffer, 0.5 μL of 10 μM forward and reverse primer, 0.12 μL RNAsin 40 U/μL, 0.5μL Superscript RT/Platinum Taq enzyme and 5μL RNA. This mixture was run using the following program for HA and M genes: 45°C for 30min, 55°C for 15min, 94°C for 2min, 30 cycles at (94°C for 45sec, 45°C for 45sec, 72°C for 5min) and 72°C for 5min. For NA gene, the following program was used: 45°C for 30min, 55°C for 15min, 94°C for 2min, 15 cycles at (94°C for 30sec, 55°C for 30sec, 72°C for 2min), 15 cycles at (94°C for 30sec, 45°C for 30sec, 72°C for 3min) and 72°C for 5min. The first PCR product was then used as a template for semi-nested PCR. A total reaction volume of 50 μL contained 38.3 μL of water, 5 μL of 10x PCR buffer, 1 μL of 10 mM dNTP mix, 1.45 mM of 50 mM MgCl_2_, 1μL of 10μM forward and reverse primer, 0.25 μL of Taq DNA polymerase 5U/μL and 2μL of first PCR product. This mixture was run using the following program for all gene segments: 94°C for 5min, 30 cycles at (94°C for 45sec, 45°C for 45sec, 72°C for 90sec) and 72°C for 5min. PCR products were run in a gel and bands corresponding to the appropriate size were sent for sequencing at GENEWIZ UK, LTD (Hope End, UK) with the corresponding primer sets.

### Phylogenetic analysis and antigenic characterization

Sequences were edited and assembled with CLC Main Workbench version 5.5. The sequences of reference strains of known clades and northern hemisphere vaccine strains recommended by WHO [18] included in phylogenetic analysis were obtained from the GenBank and GISAID databases. The phylogenetic tree of the coding nucleotide sequences was generated by MEGA version 6.0 using a neighbor-joining method. The number of bootstrap replications was set to 1000 and bootstrap values above 50% were labeled on major tree branches for reference. The amino acid residues in epitopes A to E of influenza A(H3N2) was previously identified [19].

### Nucleotide sequence accession numbers

Influenza A(H3N2) virus sequences included in the analysis were submitted to Genbank under accession numbers KY653817 to KY653919. S2 Table provides detailed information for the Cameroon influenza gene sequences.

### Prediction of glycosylation sites

The prediction of potential N-liked glycosylation sites was performed with an online server: NetNGlyc 1.0. [20]. This server considers the amino acid alignment Asn-X-Ser/Thr, where X can be any amino acid except Asp or Pro. A threshold value of >0.5 suggests glycosylation.

### Prediction of vaccine efficacy using P_epitope_ model

The predicted vaccine efficacy of the influenza A(H3N2) seasonal influenza virus was estimated using the P_epitope_ method [21–23]. P_epitope_ measures the antigenic distance between the vaccine strain and the dominant circulating strains, and it is calculated as follows: (number of mutations in the dominant epitope)/(number of amino acids in the dominant epitope). The mathematical formula linking vaccine efficacy for influenza A(H3N2) and P_epitope_ is given by E = −2.47 × P_epitope_ + 0.47 for [23]. When the P_epitope_ between the vaccine and circulating strains is null, the influenza A(H3N2) vaccine efficacy is 47% [21]. This model correlates best with vaccine efficacy as compared to phylogenetic analyses or antisera hemagglutination inhibition assay [21].

## Results

A total of 103 gene segments from the 35 virus strains were analyzed (35 HA, 35 NA and 33M). Nucleotide and amino acid sequences for the 3 selected genes were compared within the Cameroon virus strains and with the WHO vaccine strains, WHO reference strains and available sequences of other African virus strains.

### Surface glycoprotein genes and gene segments

#### Haemagglutinin

**Fig 1:**
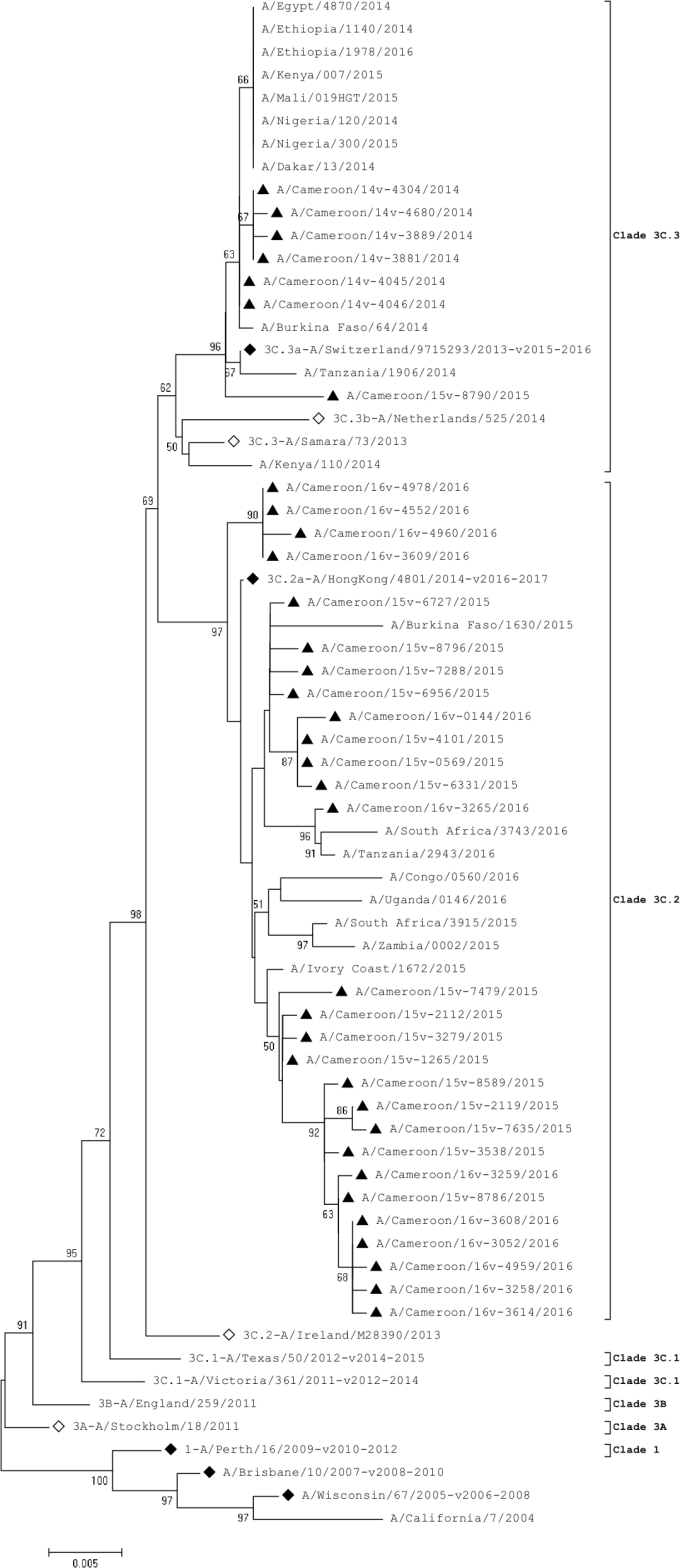
Phylogenetic analysis of HA nucleotide sequences of influenza A(H3N2). ◊ represent reference strains of known clades, ♦represent the northern hemisphere vaccine strains recommended by WHO and ▲ represent Cameroonian strains.

The HA coding sequences and amino acid sequences of the 35 Cameroonian virus strains were analyzed. Comparison of genes was performed on the strains circulating during the 2014 (N = 6), 2015 (N = 17) and 2016 (N = 12) seasons and sequences from the northern hemisphere vaccine and reference strains. Phylogenetic analysis showed that all Cameroonian virus strains had evolved away from the 3C.1-A/Texas/50/2012-like clade by acquiring several amino acid substitutions (Fig 1, Table 1). Virus strains from 2014 clustered with the 2015–2016 vaccine strain (3C.3a-A/Switzerland/9715293/2013) while virus strains from 2015 and 2016 clustered with the 2016–2017 vaccine strain (3C.2a-A/HongKong/4801/2014) except for one isolate (A/Cameroon/15V-8790/2015) which clustered with the reference strain, 3C.3a-A/Switzerland/9715293/2013.

**Table 1:** Comparison of Cameroonian virus strains with respect to A/Texas/50/2012 strain.

Antigenic sites	Amino acid substitutions		
	2014 strains	2015 strains	2016 strains
A	A138S, R142G	-	-
B	N128A, F159S	N128T, F159Y, K160T	N128T, F159Y, K160T
C	-	Q311H	Q311H

The 2014 virus strains differed from the 2015 and 2016 virus strains at three antigenic sites: antigenic site A, which contained 2 amino acid substitutions (A138S, R142G), antigenic site B showed 3 substitutions (N128T, F159Y and K160T) and antigenic site C showed a Q311H substitution when compared to the 3C.1-A/Texas/50/2012-like clade (Table 1). Isolate A/Cameroon/15V-8790/2015 differed from all other 2015 virus strains in this study by clustering in the same clade as 2014 strains. Isolate A/Cameroon/14V-4680/2014 differed from all other virus strains in this study in having a G263E substitution, and isolate A/Cameroon/16V-3265/2016 differed from all other 2016 strains by possessing N171K and R142G substitutions.

#### Neuraminidase

**Fig 2:**
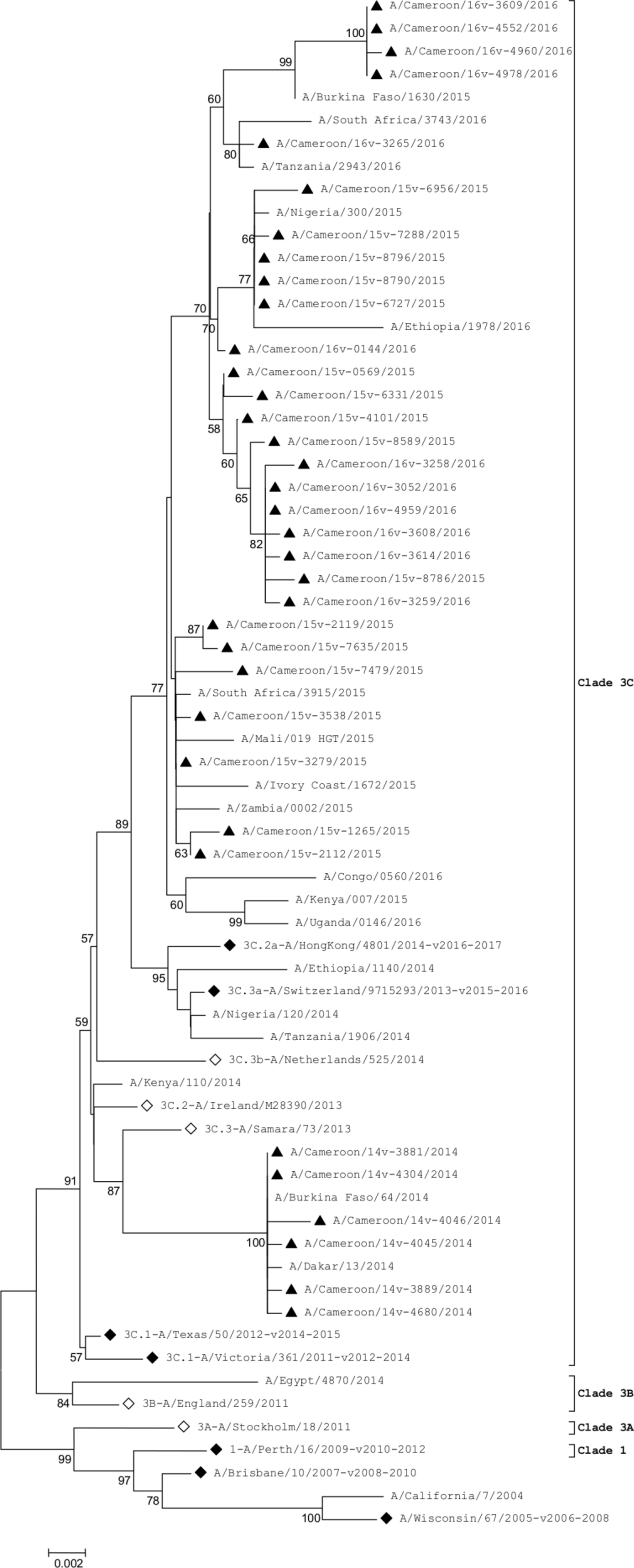
Phylogenetic analysis of NA nucleotide sequences of influenza A(H3N2). ◊ represent reference strains of known clades, ♦ represent the Northern hemisphere vaccine strains recommended by WHO and ▲ represent Cameroonian strains.

The NA sequence analysis showed that all Cameroonian strains evolved away from the 3C.1-A/Texas/50/2012-like clade (Fig 2). Strains from 2014 influenza season clustered with the 3C.3-A/Samara/73/2013 strain while 2015 and 2016 virus strains did not cluster with any strain but were closely related to the 2015–2016 and 2016–2017 vaccine strains (3C.3a-A/Switzerland/9715293/2013 and 3C.2a-A/HongKong/4801/2014, respectively).

The NA genes of the 2014 virus strains differed from those of the 2015 and 2016 virus strains by substitutions of several amino acids: E221D and T267K were found in all 2015 and 2016 virus strains. Whereas, I67V, A110D, V313A and V412I substitutions were found only in 2014 virus strains.

#### Matrix gene

**Fig 3:**
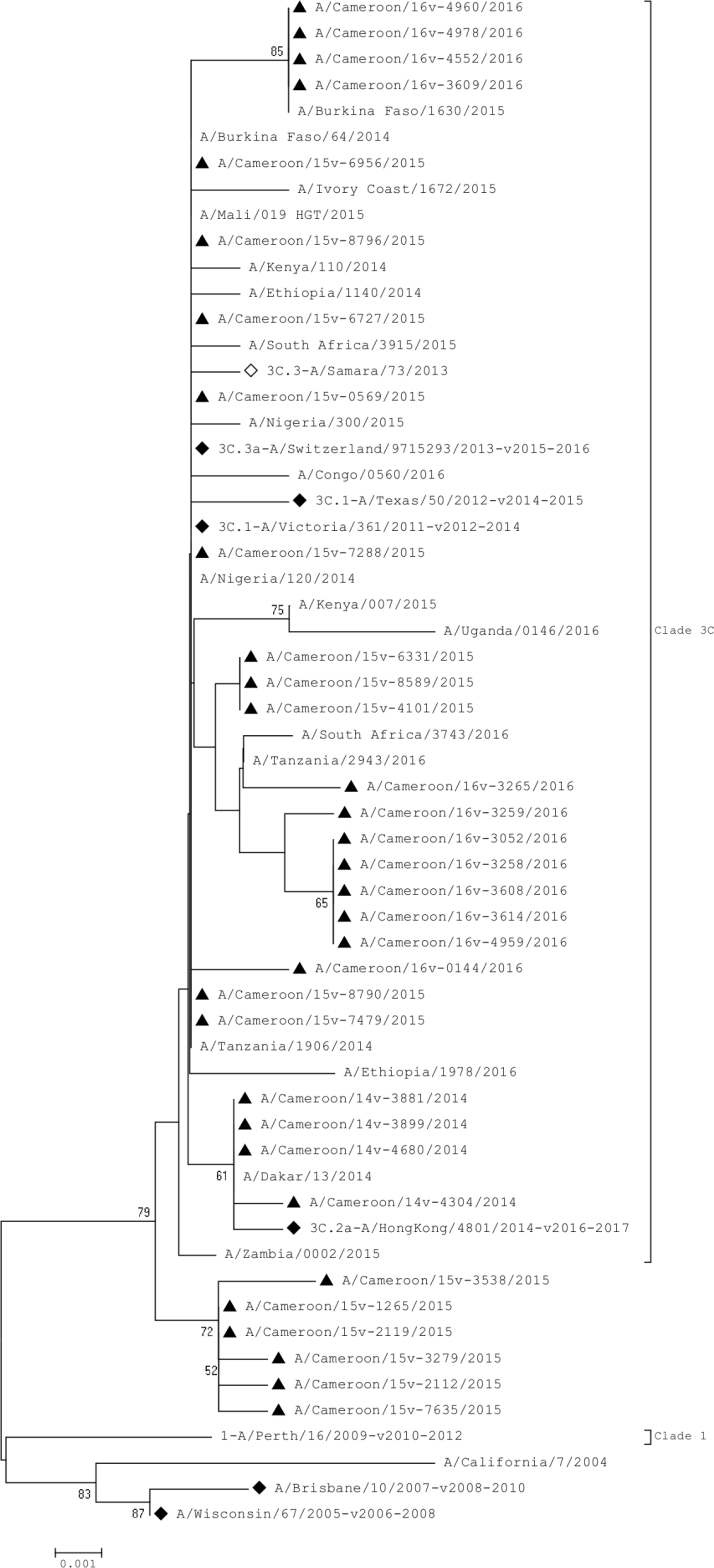
Phylogenetic analysis of M nucleotide sequences of influenza A(H3N2). **◊** represent reference strains of known clades, ♦ represent the Northern hemisphere vaccine strains recommended by WHO and ▲ represent Cameroonian strains.

Analysis of the M gene showed that 2014 virus strains clustered with the 2016–2017 vaccine strain, 3C.2a-A/HongKong/4801/2014, while 2015 and 2016 virus strains did not cluster with any reference strain (Fig 3). Globally, there was no significant change observed in the M protein of Cameroonian strains with respect to reference strains. One isolate, A/Cameroon/16V-3265/2016, displayed the amino acid substitution M165L compared to the 2016–2017 vaccine strain, A/HongKong/4801/2014.

### Genotypic antiviral drug susceptibility

In order to determine the genotypic antiviral drug susceptibility of the A(H3N2) virus strains included in this study we analyzed the NA and M2 protein coding sequences for the reported genetic markers of resistance against neuraminidase inhibitors (NAIs; oseltamivir, zanamivir, laninamivir, and peramivir) and adamantanes (amantadine and rimantadine). Mutations E41G, R118K, E119V/I/Q, Q136K (but 1 Q136H), D151A/D/E/V, R152K, I222T, R224K, Q226H, E227D, H274Y, E276D, R292K, N294S, S331R, R371K and E119V+I222L/V in the NA protein have been reported to cause reduced susceptibility to NAIs [24–27]. We laid emphasis on the following substitutions: E119I/V, 245–248 deletion, R292K, N294S and E119V+I222V that have specifically been associated with clinical resistance to neuraminidase inhibitors in A(H3N2) viruses [28–32]. None of the strains had a previously reported mutation peculiar of reduced susceptibility to NAIs however 1 isolate had an amino acid substitution at position 136 (Q136H).

Amino acids at positions 26, 27, 30 and 31 of the transmembrane region of the M2 protein are the residues frequently associated with amantadine- and rimantadine-resistant strains [33]. Amino acid sites were checked as follows for mutations associated with reduced susceptibility or resistance to adamantanes: L26F, V27A/T, A30T/V, S31N/R, G34E, A30V, S193F and R45H [25, 26]. All strains possessed the S31N amino acid substitution.

### Prediction of glycosylation sites

There were 9 predicted glycosylation sites in the HA1 of 2014 virus strains as well as one 2015 isolate (A/Cameroon/15V-8790/2015) at amino acid positions 8(NST), 22(NGT), 38(NAT), 45(NSS), 63(NCT), 133(NGT), 165(NVT), 246(NST) and 285(NGS), whereas 2015 and 2016 virus strains had 2 additional glycosylation sites at position 126(NWT) and 158(NYT) respectively due to A128T and K160T substitutions.

### Estimation of vaccine efficacy for A(H3N2)

To assess the effect of the accumulated mutations in the HA1 domain on predicted vaccine efficacy in a given year, the P_epitope_ method was used to evaluate how closely the vaccine strain resemble the circulating strain (Table 2). Theoretically, when P_epitope_ in the dominant epitope is higher than 0.19, the vaccine efficacy becomes negative [21, 22]. For 2014, the HA1 sequences mostly had a dominant mutation in epitope B (128, 159, 186, 198) and the P_epitope_ of 0.191 with respect to A/Texas/50/2012 vaccine strain, suggesting that the latter poorly matched the multiple circulating strains present that year consequently leading to a negative vaccine efficacy against these strains of -0.11% (E = -0.05% of 47%, P_epitope_ = 0). For the 2015 and 2016 seasons, HA1 sequences showed antigenic drift mainly in epitopes A. The P_epitope_ of 0.1579 for 2015 strains (dominant epitope = A; substitutions: 138, 142, 144) predicted a vaccine efficacy against these strains of 17.02% (E = 8% of 47%, P_epitope_ = 0) of that of a perfect match with the A/Switzerland/9715293/2013 vaccine strain. For 2016, P_epitope_ of 0.0526 from 9 strains (dominant epitope = A; mutation 144) predicted a minimum vaccine efficacy against these strains of 72.36% (E = 34.01% of 47%, P_epitope_ = 0) of that of a perfect match with the A/HongKong/4801/2014 vaccine strain. Influenza A(H3N2) demonstrated antigenic drift from epitopes B to epitopes A.

**Table 2:** Efficacy among the vaccine strains and number of mutations found on the dominant epitopes of influenza A(H3N2) circulating in Cameroon.

Vaccine strain	No. of strains	Dominant epitope	No. of mutations (residue differences)	P_epitope_	Predicted vaccine efficacy (%)
3C.1-A/Texas/50/2012 (2014–2015 vaccine strain)	6	B	4 (128, 159, 186, 198)	0.1905	-0.11
3C.3a-A/Switzerland/9715293/2013 (2015–2016 vaccine strain)	15	A	3 (138, 142, 144)	0.1579	17.02
	1	B	1 (198)	0.0476	74.98
	1	A	4 (135, 138, 142, 144)	0.2105	-10.62
3C.2a- A/HongKong/4801/2014 (2016–2017 vaccine strain)	9	A	1 (144)	0.0526	72.36
	1	A	1 (142)	0.0526	72.36
	1	A	2 (142, 144)	0.1053	44.66
	1	E	1 (261)	0.0455	76.09

The 2015–2016 vaccine strain A/Switzerland/9715293/2013, which belongs to clade 3C.3, was also examined for its ability to match the A(H3N2) strains of the same clade circulating in 2014. The resulting P_epitope_ value of 0 gave an estimated worst-case vaccine efficacy against these strains of 100%. Moreover, clade 3C.2 strains of A(H3N2) in 2015 season and the 2016–2017 vaccine strain, A/HongKong/4801/2014, showed a value of 0.0526, indicating a worst-case vaccine efficacy against these strains of 72.36%.

## Discussion

This is the first report of genome characterization of influenza H3N2 in Cameroon. We analyzed the coding sequences of 3 gene segments of influenza A/H3N2 virus strains from 2014 to 2016 influenza seasons in Cameroon and compared them with those of the WHO reference strains, WHO recommended vaccine strains [18]and with other epidemiologically relevant African strains from both GenBank and GISAID databases. We observed different phylogenetic clusters generated for different gene segments.

The HA gene sequences indicated that all the Cameroon strains had evolved away from the 3C.1-A/Texas/50/2012-like clade by acquiring the genetic markers N145S, Y186G, P198S and F219S which has been identified as being characteristic of clades 3C.2 and 3C.3 [13, 34]; these markers were reported in most other H3N2 influenza viruses isolated in Africa between 2014–2016. There was no cluster between the Cameroonian strains and the recommended 2014–2015 reference vaccine strain for the northern hemispheres, 3C.1-A/Texas/50/2012-like clade. None of the selected African virus strains circulating between 2014 and 2016 clustered in the 3C.1-A/Texas/50/2012 clade either. Cameroonian strains formed two distinct clusters: 2014 virus strains clustered with the 3C.2a-A/Switzerland/9715293/2013-like clade while the 2015 and 2016 virus strains clustered with the 3C.2a-A/Hong Kong/4801/2014-like clade. Interestingly, the Cameroonian NA gene sequences had also evolved away from the 3C.1-A/Texas/50/2012-like clade by acquiring several amino acid substitutions. A unique 2015 isolate, A/Cameroon/15V-8790/2015, was comparable in many regards to the 2014 virus strains; however, its M and NA genes were unlike the remaining 2015 virus strains.

Theoretically, a clear H3N2 vaccine mismatch was observed during the 2014–2015 influenza seasons as reported by Tewawong *et al*. in the Southern hemisphere [22]. Several amino acid mutations on epitope A of the HA gene were responsible for the drift between the circulating strains and the 2014–2015 vaccine strains, 3C.1-A/Texas/50/2012-like strain. This corroborates with the P_epitope_ > 0.19 observed, and eventually resulting in a negative vaccine efficacy. The vaccine change to the A/Switzerland/9715293/2013-like strain for the following season resulted in more than 80% of the circulating H3N2 strains not covered by the selected vaccine. Notwithstanding the poor results with the previous influenza seasons, the 2016–2017 selected vaccine strain showed moderate efficacy when compared to the circulating virus strains. It is noteworthy that vaccination against influenza virus, is not a common phenomenon in the Cameroon population, however a better selection of annual vaccine with the use of P_epitope_ as suggested by other authors, [21] will improve vaccine efficacy and encourage uptake of flu vaccine. This may result in reduced burden caused by influenza due to herd immunity [35].

The acquisition of a mutation in influenza A viruses conferring resistance to an antiviral agent may occur as a result of drug selection, spontaneous mutation or through genetic reassortment with another drug resistant influenza A virus [26]. Neuraminidase inhibitors target the NA glycoprotein which plays a key role in the production of new viral progeny. There have been rare reports of resistance to neuraminidase inhibitors in influenza H3N2 virus strains [27], and this was confirmed Cameroonian virus strains by the absence of previous mutations characteristic of this resistance. There was however a novel amino an amino acid mutation, Q136H, that had not been previously reported. Further investigation on the clinical relevance of this mutation will be essential for a better understanding of the molecular evolution of influenza A(H3N2).

Amantadine resistance reported in most H3N2 viruses worldwide was confirmed in the Cameroonian virus strains by an S31N substitution in the M2 protein [25, 27]. High prevalence of amantadine resistance in influenza A viruses was observed in other countries, irrespective of its use [25, 26]. Resistance to M2 inhibitors first appeared following extensive drug use in Asia and the US after the SARS (Severe Acute Respiratory Syndrome) epidemic in 2004. Since there is no history of exposure to anti-viral drugs against influenza and eventually to adamantanes, Cameroonian virus strains with S31N mutation indicated that strains already harbouring drug resistant mutation were most likely introduced in the community. These resistant strains probably have a selective advantage over the sensitive strains that favour their evolution and fitness [36].

As reported by Mostafa *et al*., glycosylation and deglycosylation are important viral mechanisms to (i) mask antigenic epitopes and thus inhibit binding to neutralizing antibodies, (ii) adjust receptor-binding affinity, or (iii) regulate virulence of influenza [14]. The Cameroonian H3N2 virus strains shared at least nine potential N-glycosylation sites with the 2014–2017 vaccine strains, and had similar glycosylation patterns. Group 3C.2a-like strains had acquired 2 additional glycosylation sites: 126NWT128 and 158NYT160. The predicted HA glycosylation patterns may contribute to a potential antigenic escape of influenza H3N2 viruses from antibodies raised against vaccine strain and previous circulating strains or increase viral fitness by yet-to-be-characterized mechanisms.

## Conclusion

The presence of several antigenic site mutations with high frequency among H3N2 virus strains between 2014–2016 influenza seasons confirms the progressing evolution of circulating H3N2 strains. These results lay more emphasis on the relevance of routine influenza surveillance as well as the genetic and antigenic characterization in facilitating the prompt and efficient selection of the influenza strains which will be included in the flu vaccine. This data will be very valuable in tracking the phylogenetic evolution of influenza viruses within sub-Saharan Africa.

## Supporting information

S1 Table Set of primers for PCR and sequencing. (PDF)

S2 Table Identification of Cameroon H3N2 virus strains. (PDF)
