# Supplementary material for: Genetic and antigenic characterization of influenza A(H3N2) in Cameroon during the 2014-2016 influenza seasons
Source: PLoS One. 2017 Sep 6;12(9):e0184411. doi: 10.1371/journal.pone.0184411 (PMC5587321; doi:10.1371/journal.pone.0184411)
Supplement: S1 Table — (PDF) [file pone.0184411.s001.pdf]

Gene	Oligo name	Primer sequences 5'-3'
Hemagglutinin (H3)	H3/+/6B	AAGCAGGGGATAATTCTATTAACC
	H3/+/361	GCAACTGTTACCCTTATGATGTG
	H3/-/694	GAGACTGTGACTCTCCCTGATG
	H3/-/1197	CTGCTTGAGTGCTTTTAAGATCTG
Neuraminidase (N2)	A/N2/+/1	AGCAAAAGCAGGAGTGAAGATG
	A/N2/+/823	GTCAGGAAGTGCTCAGCATGTAG
	A/N2/-/972	GCACACATAACTGGAATCAATGC
	A/N2/-1459	CAAGGAGTTTTTTTCTAAAATTGCG
Matrix (M)	1Mf (1F)	AGCAAAAGCAGGTAGATATTGA
	1MRC (1027R)	AGTAGAAACAAGGTAGTTTTTTACTC
	2Mf (237F)	AGCGAGGACTGCAGCGTAG
	2MRC (841R)	GAATCCACAATATCAAGTGCA
