# Supplementary material for: Genetic and antigenic characterization of influenza A(H3N2) in Cameroon during the 2014-2016 influenza seasons
Source: PLoS One. 2017 Sep 6;12(9):e0184411. doi: 10.1371/journal.pone.0184411 (PMC5587321; doi:10.1371/journal.pone.0184411)
Supplement: S2 Table — (PDF) [file pone.0184411.s002.pdf]

Organism	Accession No.	Collection date	Segment
A/Cameroon/14V-3881/2014	KY653817	9-Jun-2014	HA
A/Cameroon/14V-3881/2014	KY653818	9-Jun-2014	NA
A/Cameroon/14V-3881/2014	KY653819	9-Jun-2014	M
A/Cameroon/14V-3889/2014	KY653820	6-Jun-2014	HA
A/Cameroon/14V-3889/2014	KY653821	6-Jun-2014	NA
A/Cameroon/14V-3889/2014	KY653822	6-Jun-2014	M
A/Cameroon/14V-4045/2014	KY653823	3-Jun-2014	HA
A/Cameroon/14V-4045/2014	KY653824	3-Jun-2014	NA
A/Cameroon/14V-4046/2014	KY653825	5-Jun-2014	HA
A/Cameroon/14V-4046/2014	KY653826	5-Jun-2014	NA
A/Cameroon/14V-4304/2014	KY653827	20-Jun-2014	HA
A/Cameroon/14V-4304/2014	KY653828	20-Jun-2014	NA
A/Cameroon/14V-4304/2014	KY653829	20-Jun-2014	M
A/Cameroon/14V-4680/2014	KY653830	4-Jul-2014	HA
A/Cameroon/14V-4680/2014	KY653831	4-Jul-2014	NA
A/Cameroon/14V-4680/2014	KY653832	4-Jul-2014	M
A/Cameroon/15V-0569/2015	KY653833	14-Jan-2015	HA
A/Cameroon/15V-0569/2015	KY653834	14-Jan-2015	NA
A/Cameroon/15V-0569/2015	KY653835	14-Jan-2015	M
A/Cameroon/15V-1265/2015	KY653836	5-Feb-2015	HA
A/Cameroon/15V-1265/2015	KY653837	5-Feb-2015	NA
A/Cameroon/15V-1265/2015	KY653838	5-Feb-2015	M
A/Cameroon/15V-2112/2015	KY653839	9-Mar-2015	HA
A/Cameroon/15V-2112/2015	KY653840	9-Mar-2015	NA
A/Cameroon/15V-2112/2015	KY653841	9-Mar-2015	M
A/Cameroon/15V-2119/2015	KY653842	12-Mar-2015	HA
A/Cameroon/15V-2119/2015	KY653843	12-Mar-2015	NA
A/Cameroon/15V-2119/2015	KY653844	12-Mar-2015	M
A/Cameroon/15V-3279/2015	KY653845	17-Apr-2015	HA
A/Cameroon/15V-3279/2015	KY653846	17-Apr-2015	NA
A/Cameroon/15V-3279/2015	KY653847	17-Apr-2015	M
A/Cameroon/15V-3538/2015	KY653848	5-May-2015	HA
A/Cameroon/15V-3538/2015	KY653849	5-May-2015	NA
A/Cameroon/15V-3538/2015	KY653850	5-May-2015	M
A/Cameroon/15V-4101/2015	KY653851	18-May-2015	HA

A/Cameroon/15V-4101/2015	KY653852	18-May-2015	NA
A/Cameroon/15V-4101/2015	KY653853	18-May-2015	M
A/Cameroon/15V-6331/2015	KY653854	26-Aug-2015	HA
A/Cameroon/15V-6331/2015	KY653855	26-Aug-2015	NA
A/Cameroon/15V-6331/2015	KY653856	26-Aug-2015	M
A/Cameroon/15V-6727/2015	KY653857	17-Sep-2015	HA
A/Cameroon/15V-6727/2015	KY653858	17-Sep-2015	NA
A/Cameroon/15V-6727/2015	KY653859	17-Sep-2015	M
A/Cameroon/15V-6956/2015	KY653860	23-Sep-2015	HA
A/Cameroon/15V-6956/2015	KY653861	23-Sep-2015	NA
A/Cameroon/15V-6956/2015	KY653862	23-Sep-2015	M
A/Cameroon/15V-7288/2015	KY653863	8-Oct-2015	HA
A/Cameroon/15V-7288/2015	KY653864	8-Oct-2015	NA
A/Cameroon/15V-7288/2015	KY653865	8-Oct-2015	M
A/Cameroon/15V-7479/2015	KY653866	7-Oct-2015	HA
A/Cameroon/15V-7479/2015	KY653867	7-Oct-2015	NA
A/Cameroon/15V-7479/2015	KY653868	7-Oct-2015	M
A/Cameroon/15V-7635/2015	KY653869	23-Oct-2015	HA
A/Cameroon/15V-7635/2015	KY653870	23-Oct-2015	NA
A/Cameroon/15V-7635/2015	KY653871	23-Oct-2015	M
A/Cameroon/15V-8589/2015	KY653872	18-Nov-2015	HA
A/Cameroon/15V-8589/2015	KY653873	18-Nov-2015	NA
A/Cameroon/15V-8589/2015	KY653874	18-Nov-2015	M
A/Cameroon/15V-8786/2015	KY653875	17-Nov-2015	HA
A/Cameroon/15V-8786/2015	KY653876	17-Nov-2015	NA
A/Cameroon/15V-8786/2015	KY653877	17-Nov-2015	M
A/Cameroon/15V-8790/2015	KY653878	25-Nov-2015	HA
A/Cameroon/15V-8790/2015	KY653879	25-Nov-2015	NA
A/Cameroon/15V-8790/2015	KY653880	25-Nov-2015	M
A/Cameroon/15V-8796/2015	KY653881	1-Dec-2015	HA
A/Cameroon/15V-8796/2015	KY653882	1-Dec-2015	NA
A/Cameroon/15V-8796/2015	KY653883	1-Dec-2015	M
A/Cameroon/16V-0144/2016	KY653884	6-Jan-2016	HA
A/Cameroon/16V-0144/2016	KY653885	6-Jan-2016	NA
A/Cameroon/16V-0144/2016	KY653886	6-Jan-2016	M
A/Cameroon/16V-3052/2016	KY653887	22-Apr-2016	HA

A/Cameroon/16V-3052/2016	KY653888	22-Apr-2016	NA
A/Cameroon/16V-3052/2016	KY653889	22-Apr-2016	M
A/Cameroon/16V-3258/2016	KY653890	27-Apr-2016	HA
A/Cameroon/16V-3258/2016	KY653891	27-Apr-2016	NA
A/Cameroon/16V-3258/2016	KY653892	27-Apr-2016	M
A/Cameroon/16V-3259/2016	KY653893	28-Apr-2016	HA
A/Cameroon/16V-3259/2016	KY653894	28-Apr-2016	NA
A/Cameroon/16V-3259/2016	KY653895	28-Apr-2016	M
A/Cameroon/16V-3265/2016	KY653896	4-May-2016	HA
A/Cameroon/16V-3265/2016	KY653897	4-May-2016	NA
A/Cameroon/16V-3265/2016	KY653898	4-May-2016	M
A/Cameroon/16V-3608/2016	KY653899	17-May-2016	HA
A/Cameroon/16V-3608/2016	KY653900	17-May-2016	NA
A/Cameroon/16V-3608/2016	KY653901	17-May-2016	M
A/Cameroon/16V-3609/2016	KY653902	18-May-2016	HA
A/Cameroon/16V-3609/2016	KY653903	18-May-2016	NA
A/Cameroon/16V-3609/2016	KY653904	18-May-2016	M
A/Cameroon/16V-3614/2016	KY653905	16-May-2016	HA
A/Cameroon/16V-3614/2016	KY653906	16-May-2016	NA
A/Cameroon/16V-3614/2016	KY653907	16-May-2016	M
A/Cameroon/16V-4552/2016	KY653908	8-Jun-2016	HA
A/Cameroon/16V-4552/2016	KY653909	8-Jun-2016	NA
A/Cameroon/16V-4552/2016	KY653910	8-Jun-2016	M
A/Cameroon/16V-4959/2016	KY653911	14-Jun-2016	HA
A/Cameroon/16V-4959/2016	KY653912	14-Jun-2016	NA
A/Cameroon/16V-4959/2016	KY653913	14-Jun-2016	M
A/Cameroon/16V-4960/2016	KY653914	10-Jun-2016	HA
A/Cameroon/16V-4960/2016	KY653915	10-Jun-2016	NA
A/Cameroon/16V-4960/2016	KY653916	10-Jun-2016	M
A/Cameroon/16V-4978/2016	KY653917	8-Jun-2016	HA
A/Cameroon/16V-4978/2016	KY653918	8-Jun-2016	NA
A/Cameroon/16V-4978/2016	KY653919	8-Jun-2016	M
